# Using Whole Genome Sequencing to Trace, Control and Characterize a Hospital Infection of IMP-4-Producing *Klebsiella pneumoniae* ST2253 in a Neonatal Unit in a Tertiary Hospital, China

**DOI:** 10.3389/fpubh.2021.755252

**Published:** 2021-12-15

**Authors:** Yuanyuan Bai, Chunhong Shao, Yingying Hao, Yueling Wang, Yan Jin

**Affiliations:** Department of Clinical Laboratory, Shandong Provincial Hospital Affiliated to Shandong First Medical University, Jinan, China

**Keywords:** *Klebsiella pneumoniae*, whole genome sequencing, drug resistance, carbapenem, single nucleotide polymorphisms

## Abstract

**Background:** The purpose of this study is to use whole genome sequencing (WGS) combined with epidemiological data to track a hospital infection of the carbapenem-resistant *Klebsiella pneumoniae* (CRKP), which affected 3 neonatal patients in the neonatal intensive care unit (NICU).

**Methods:** The minimum inhibitory concentrations for the antimicrobial agents were determined according to the guidelines of the Clinical and Laboratory Standards Institute. Beta-lactamases were investigated using the polymerase chain reaction and DNA sequencing. The transferability of the plasmid was investigated by a conjugation experiment. The clonal relationships were evaluated using multilocus sequence typing (MLST) and pulsed-field gel electrophoresis (PFGE). WGS and single nucleotide polymorphisms (SNPs) analysis were performed on the CRKP isolates to investigate how the infection might progress.

**Results:** Nine CRKP isolates were obtained from the NICU, seven from three patients, one from a duster cloth and one from the hand of a nurse, they all harbored *bla*IMP-4. Other resistance genes including *bla*KPC-2, *bla*IMP-4, *bla*SHV-1, *bla*TEM-1, *bla*CTX-M-15, and *bla*DHA-1 were also detected. PFGE analysis showed that IMP-4-producing *K. pneumoniae* were clonally related, and MLST assigned them to a new sequence type 2253. The SNP variations throughout the genome divided the 9 strains into three clades. Clade 1 comprised 7 strains (K1- K2 and K4-K8), whereas clade 2 and 3 consisted of only one strain each: K3 and K9, respectively.The sputum isolate K3 from patient 3 was the most distinct one differing from the other eight isolates by 239-275 SNPs.

**Conclusions:** This is a report of using WGS to track a hospital infecion of IMP-4-producing *K. pneumoniae* ST2253 among neonates. Nosocomial surveillance systems are needed to limit the spread of the infection caused by these pathogens resulting from the environmental exposure in NICUs.

## Introduction

Carbapenem-resistant *Klebsiella pneumoniae* (CRKP), which was mainly mediated by plasmid-mediated carbapenemase (1), has become a major public health threat. Three families of acquired carbapenemase were reported, including two major types: the molecular class A *K. pneumoniae* carbapenemase (KPCs) and the class B metallo-β-lactamases (MBLs) (2–4). The KPC-types, especially KPC-2, and MBLs, including IMP, VIM families and NDM-1, were found to be harbored by non-fermentative bacteria and by members of the *Enterobacteriaceae* worldwide (5–9). MBLs are the most clinically threatening carbapenems due to their ability to hydrolyze almost all β-lactams except for aztreonam and the fact that they are not inhibited by therapeutic β-lactams inhibitors such as clavulanate, sulbactam, avibactam, and tazobactam.

The *bla*IMP-4 gene, which encodes IMP-4 MBL, was first identified in the late 1990s in *Acinetobacter* spp. from Hong Kong (10) and *Citrobacter youngae* from Guangzhou in China and caused an outbreak in 2005 in Australia (11). The *bla*IMP-4 gene was usually found to co-exist with other carbapenemase resistance-genes in *K. pneumoniae* isolates. KPC has emerged as the predominant carbapenemase in *K. pneumoniae* in China, while there are relatively few IMP-4 producing strains (8, 9, 12), and an outbreak caused by CRKP harboring *bla*IMP-4 in neonates remains uncommon in China.

With the rapid technological advances, whole genome sequencing (WGS) has emerged as a powerful tool for bacterial typing (13, 14). The success in tracking worldwide epidemics (15–18), regional outbreaks (16, 19) and foodborne outbreaks (20, 21) has demonstrated that the fine resolution of WGS can facilitate our understanding of how infectious agents spread.

In this study, we observed nine CRKP sourced from patients in the neonatal intensive care unit (NICU) and applied WGS to source tracing a hospital infection of CRKP in NICU.

## Materials and Methods

### Bacterial Isolates Collected in This Study

From November 9 to November 13, 2018, three preterm infants were admitted to the NICU of the Shandong Provincial Hospital, which is affiliated to Shandong First Medical University. Seven *K. pneumoniae* isolates were obtained from different clinical specimens of the three patients. Environmental sampling was performed in the patient's ward using sponge swabs and subsequent culture was performed in brain heart infusion (BHI) mediums for 24 h. Two *K. pneumoniae* isolates obtained from the environmental screening were included in this study.

### Antimicrobial Susceptibility Testing, Carbapenemase Inactivation Methods and EDTA Synergy Tests

The antimicrobial susceptibilities were determined using the GNS cards of the Vitek system (bioMérieux). The tested antibiotics included were listed in Table 1. The minimum inhibitory concentrations (MICs) results were interpreted as specified by the, Clinical and Laboratory Standards Institute (CLSI) 2018 criteria, and the breakpoints of tigecycline MIC were determined following the guidelines of the U.S. Food and Drug Administration (MIC ≤ 2 mg/L denoting susceptibility and MIC ≥8 mg/L denoting resistance). *E. coli* ATCC 25922 was used as a quality control strain for the antimicrobial susceptibility testing. The production of carbapenemase was evaluated in all the isolates using the carbapenem inactivation method (CIM) (22). EDTA was used to screen for class A and class B carbapenemases.

**Table 1:** Drug resistance profiles of IMP-4-producing *K. pneumonia* and *E.coli* J53 transconjugant strains derived from IMP-4-producing *K. pneumonia* transconjugants.

**Strains**	**MIC**											
	**IPM**	**MEM**	**ETP**	**TGC**	**ATM**	**AK**	**GEN**	**FEP**	**FOX**	**CAZ**	**CRO**	**SXT**
K1	32 (R)	32 (R)	32 (R)	2 (S)	≤1 (S)	≤2 (S)	≤1 (S)	8 (SDD)	32 (R)	≥64 (R)	≥64 (R)	≥16 (R)
K2	32 (R)	32 (R)	32 (R)	1 (S)	≤1 (S)	≤2 (S)	≤1 (S)	8 (SDD)	32 (R)	≥64 (R)	≥64 (R)	≥16 (R)
K3	32 (R)	32 (R)	32 (R)	1 (S)	≤1 (S)	≤2 (S)	≤1 (S)	8 (SDD)	32 (R)	≥64 (R)	≥64 (R)	≥16 (R)
K4	32 (R)	32 (R)	32 (R)	1 (S)	≤1 (S)	≤2 (S)	≤1 (S)	8 (SDD)	32 (R)	≥64 (R)	≥64 (R)	≥16 (R)
K5	32 (R)	32 (R)	32 (R)	1.5 (S)	≤1 (S)	≤2 (S)	≤1 (S)	8 (SDD)	32 (R)	≥64 (R)	≥64 (R)	≥16 (R)
K6	32 (R)	32 (R)	32 (R)	2 (S)	≤1 (S)	≤2 (S)	≤1 (S)	8 (SDD)	32 (R)	≥64 (R)	≥64 (R)	≥16 (R)
K7	32 (R)	32 (R)	32 (R)	1.5 (S)	≤1 (S)	≤2 (S)	≤1 (S)	8 (SDD)	32 (R)	≥64 (R)	≥64 (R)	≥16 (R)
K8	32 (R)	32 (R)	16 (R)	1.5 (S)	≤1 (S)	≤2 (S)	≤1 (S)	8 (SDD)	32 (R)	≥64 (R)	≥64 (R)	≥16 (R)
K9	32 (R)	32 (R)	32 (R)	1 (S)	≤1 (S)	≤2 (S)	≤1 (S)	8 (SDD)	32 (R)	≥64 (R)	≥64 (R)	≥16 (R)
K1-J53	8 (R)	8 (R)	8 (R)	≤0.5 (S)	≤1 (S)	≤2 (S)	≤1 (S)	8 (SDD)	≥64 (R)	≥64 (R)	≥64 (R)	4 (R)
K2-J53	8 (R)	8 (R)	8 (R)	≤0.5 (S)	≤1 (S)	≤2 (S)	≤1 (S)	8 (SDD)	≥64 (R)	≥64 (R)	≥64 (R)	≥16 (R)
K3-J53	8 (R)	8 (R)	8 (R)	≤0.5 (S)	≤1 (S)	≤2 (S)	≤1 (S)	8 (SDD)	≥64 (R)	≥64 (R)	≥64 (R)	≥16 (R)
K4-J53	8 (R)	16 (R)	1 (I)	≤0.5 (S)	≤1 (S)	≤2 (S)	≤1 (S)	8 (SDD)	≥64 (R)	≥64 (R)	≥64 (R)	4 (R)
K5-J53	8 (R)	8 (R)	1 (I)	≤0.5 (S)	≤1 (S)	≤2 (S)	≤1 (S)	16 (R)	≥64 (R)	≥64 (R)	≥64 (R)	4 (R)
K6-J53	8 (R)	8 (R)	1 (I)	≤0.5 (S)	≤1 (S)	≤2 (S)	≤1 (S)	4 (SDD)	≥64 (R)	≥64 (R)	≥64 (R)	4 (R)
K7-J53	2 (I)	8 (R)	1 (I)	≤0.5 (S)	≤1 (S)	≤2 (S)	≤1 (S)	4 (SDD)	≥64 (R)	≥64 (R)	≥64 (R)	4 (R)
K8-J53	2 (I)	>32 (R)	1 (I)	≤0.5 (S)	≤1 (S)	≤2 (S)	≤1 (S)	4 (SDD)	≥64 (R)	≥64 (R)	≥64 (R)	4 (R)
K9-J53	2 (I)	>32 (R)	2 (R)	≤0.5 (S)	≤1 (S)	≤2 (S)	≤1 (S)	4 (SDD)	≥64 (R)	≥64 (R)	≥64 (R)	≥16 (R)
EC J53	<1 (S)	<1 (S)	<0.5 (S)	0.25 (S)	<1 (S)	<2 (S)	<1 (S)	<1 (S)	<1 (S)	<1 (S)	<1 (S)	<0.5 (S)

*MIC, Minimum inhibitory concentration; CRO, Ceftriaxone; CAZ, ceftazidime; FEP, cefepime; FOX, cefoxitin; SXT, trimethoprim-sulfamethoxazole; MEM, meropenem; IMP, imipenem; ETP, ertapenem; GEN, gentamicin; AK, amikacin; ATM, aztreonam; TGC, tigecycline; EC J53, recipient*.

### Detection of Drug-Resistant Genes

The bacterial genome DNA was obtained from the clinical strains. Next, PCR and DNA sequence analysis were performed to confirm the presence of drug-resistant genes. The primers that were used in this study were previously described (23). The β-lactamase genes, including Ambler class A (*bla*CTX-M, *bla*TEM, *bla*SHV, *bla*KPC, *bla*IMI and *bla*GES), class B (*bla*VIM, *bla*IMP, *bla*NDM, and *bla*SPM) and class C (*bla*CMY, *bla*ACT-1, and *bla*DHA-1) were investigated in all the clinical isolates. The PCR products were then checked on 1% agarose electrophoresis gels, stained with ethidium bromide and visualized under UV light. Finally, the products were sequenced and compared with the reported sequences from GenBank.

### PFGE and MLST

The IMP-4-producing strains were genotyped using MLST and PFGE. The genomic DNA was prepared from all the tested CRKP isolates and cleaved with XbaI.

The detailed methods referred to Jin et al. (24) Minimum spanning tree was constructed using PHYLOViZ (https://online.phyloviz.net/index) online software.

### Conjugation Experiments

In order to determine whether the carbapenem resistance was transferable in the *K. pneumoniae* isolates, we carried out a conjugation experiment in Luria–Bertani (LB) broth using *E. coli* J53 as the recipient, as previously described (25).

### Whole Genome Sequencing

The total DNA was extracted from the 9 IMP-4-producing *K. pneumoniae* strains and sequenced using the next generation sequencing (NGS) technology. Whole genome sequencing was performed on the Illumina HiSeq PE150 platformand a long-read MinION sequencer (Nanopore, Oxford, UK) were used for whole-genome sequencing. The *de novo* hybrid assembly of both short Illumina reads and longMinION reads were performed using Unicycler (26). Comparing the draft genome sequence with the complete *K. pneumoniae* genomes in GenBank indicated that the isolate K7, which was selected as the reference genome, was closely related to each clinical strain, and could thus be used as the reference genome.

### Single Nucleotide Polymorphisms and Phylogenetic Tree

In order to detect the SNPs between the reference genome and the other eight clinical strains, the paired-end reads of each strain were directly mapped to the chromosome of the K1 strain using the CLC Genomics Workbench 7.0 (CLC bio, Aarhus, Denmark). The high-quality SNPs (mapping coverage of >30) were selected and ranked in a row based on their relative order to the reference genome. Next, a core genome phylogenetic tree was constructed, using a bootstrap value of 1,000.

### Accession Codes

The whole genome sequences of the nine IMP-producing *K. pneumoniae* strains were deposited in the Short Read Archive (SRA) with the accession numbers SRR8607308 (K1), SRR8607311 (K2), SRR8607306 (K3), SRR8607305 (K4), SRR8607309 (K5), SRR8607310 (K6), SRR8607307 (K7), SRR8607313 (K8), SRR8607312 (K9), respectively.

## Results

### Infection Description

**Figure 1:**
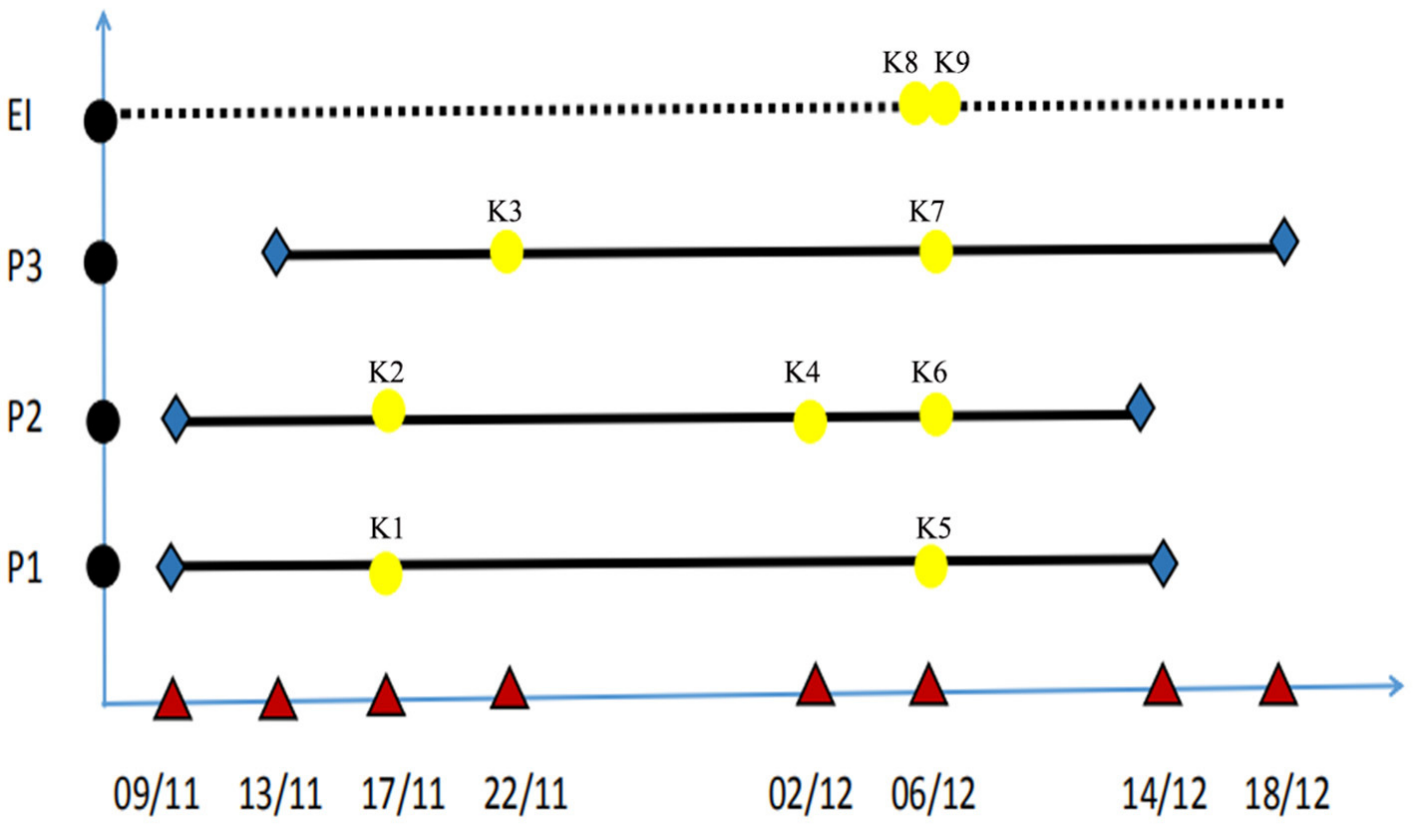
Temporal graph of the nine isolates of ST2253 isolated from three patients and the environment of NICU (K1–K9). The used symbols are as following: Black circles, the patients and environment; P, patient; EN, environment; Red triangle, date; Yellow, detection time; Blue, admission time and discharge time; Bold black line, period of hospitalization.

Seven CRKP strains (K1–K7) were isolated from three patients from a variety of specimens including sputum, rectal swabs and blood. Due to neonates are not able to produce sputum, sputum specimens were obtained by endotrachea sputum-suction method. On November 17, 2018, two *K. pneumoniae* strains (K1, K2) were isolated from the sputum specimens that were, respectively, obtained from two neonatal twin patients (patient 1 and patient 2) in the NICU. The two strains were both resistant to carbapenems including meropenem. On November 22, 2018, the third CRKP strain (K3) was isolated from a sputum sample that was obtained from another neonate (patient 3) in the same ward. On December 02, 2018, the fourth CRKP (K4) was isolated from a blood sample from patient 2. After the CRKP strain was isolated from the third neonate, we organized an nosocomial infection control team and immediately implemented the infection control measures. We screened the rectal swab samples taken from the three patients in the NICU ward and obtained rectal swabs and breast milk samples from their mothers. CRKP strains K5, K6, and K7 were isolated from the rectal swabs of the three patients, respectively. Environmental samples of the bed linen, inside of the neonatal incubator, duster cloth, doorknobs and the hand swabs were simultaneously obtained from the doctors and nurses. The bacterial isolates were identified by a Vitek-2 Compact (bioMérieux) according to the manufacturer's instructions and additional biochemical tests. Two CRKP isolates (K8, K9) were recovered from the environmental samples, comprising one from the hand of a nurse and one from a duster cloth in the ward. The temporal graph of the nine strains that were isolated from the three patients and the environment of the ward is shown in Figure 1. The gestational ages of the 3 patients ranged from 29 to 31 weeks, and their weights were only 1,320–1,510 g, both of these represent risk factors for the acquisition of hospital-associated infections. The CRKP infection was declared on November 17, 2018.

### Drug-Resistant Genes and Antimicrobial Susceptibility Testing

The PCR analysis using specific primers for the genes of *bla*KPC, *bla*NDM, *bla*IMP, *bla*VIM, *bla*SHV, and *bla*DHA followed by the nucleotide sequencing of both DNA strands revealed the simultaneous presence of *bla*IMP-4 genes. All the strains (K1–K9) harbored *bla*IMP-4, which is a carbapenemase-encoding gene. The MBL screening test results were positive by CIM and EDTA synergy test.

The drug-resistance profiles were consistent between the seven IMP-4-producing *K. pneumoniae* clinical isolates (K1–K7) and the two IMP-4-producing *K. pneumoniae* strains obtained from the environment (K8, K9). All the nine strains were highly resistant to the tested carbapenems, including meropenem. All the strains were susceptible to aztreonam. The MIC values for the other tested β-lactam antibiotics were high (≥64 μg/mL) in all the tested strains. Tigecycline exhibited a potent activity against all the tested strains, no tigecycline resistant strains were thus detected. All the isolates remained susceptible to levofloxacin, amikacin and gentamicin. These results are summarized in Table 1.

### PFGE and MLST Typing

**Figure 2:**
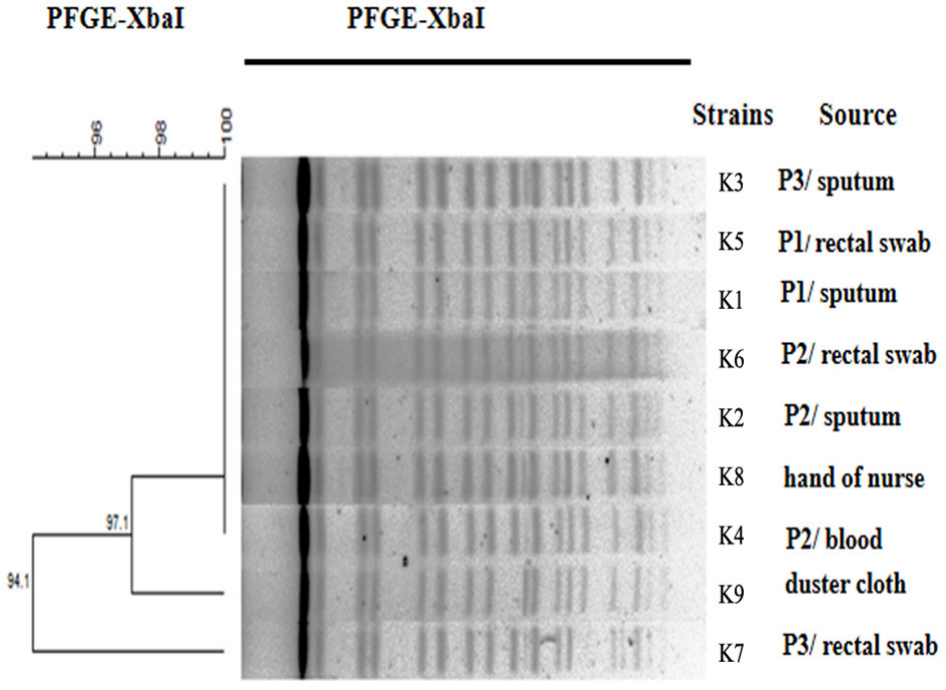
Dendrogram analysis. Dendrogram generated using the Fingerprinting II Informatix software package (Bio-Rad Laboratories, Hercules, CA) showing the relatedness of the fingerprints (XbaI-PFGE) for the 9 *K. pneumoniae* strains. The phylogenetic tree was constructed using the Dice coefficient and UPGMA clustering. A genetic similarity index scale is shown to the left of the dendrogram. P, patient.

The PFGE patterns of the XbaI DNA digests of the 9 *K. pneumoniae* isolates were obtained. These patterns exhibited high similarity between the nine isolates (Figure 2). MLST has indeed assigned both of these isolates to a novel sequence type, namely ST2253 (*gapA*-4, *infB*-5, *mdh*-2, *pgi*-2, *phoE*-1, *rpoB*-1, *tonB*-24).

The novel ST2253 was compared with the main ST types of IMP-4 producing CR-KPs that had been reported in China by phylogenetic tree (Supplementary Figure 1).

### Bacterial Conjugation

The 9 IMP-4-positive *K. pneumoniae* isolates were selected to perform conjugation experiments. The results indicated that the plasmids with *bla*IMP-4 were successfully transferred from all the *K. pneumoniae* isolates to the recipient *E. coli* J53, The MIC values of the nine transconjugants were tested, and all the *E. coli* transconjugants exhibited a significantly reduced carbapenem susceptibility to the tested carbapenems compared with the *E. coli* J53 (Table 1). The nine CRKP strains all carried the IncN plasmid.

### WGS and SNPs Analysis

**Figure 3:**
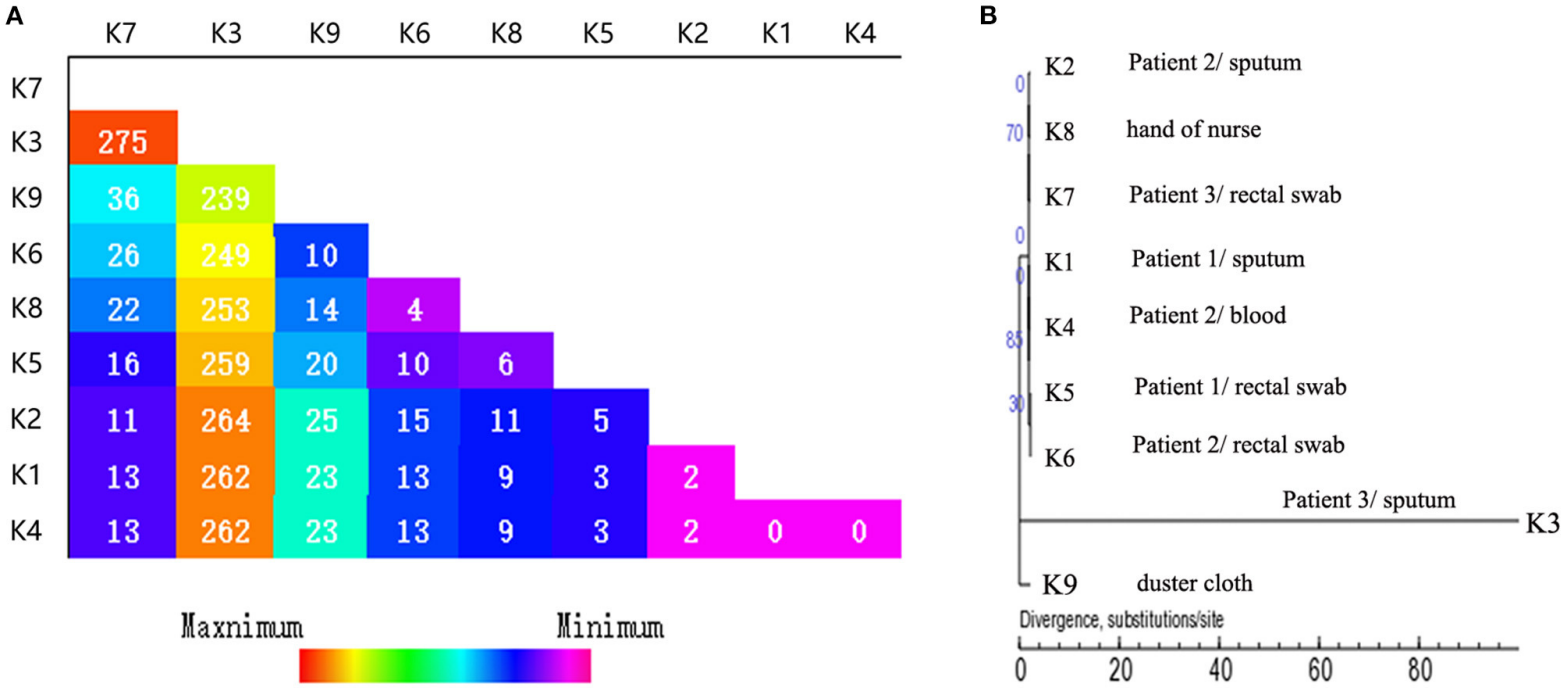
WGS and SNPs analysis. **(A)** The single nucleotide polymorphisms (SNPs) numbers between each IMP-4-producing strain and the reference strain. **(B)** The phylogenetic analysis of the nine CRKP isolates based on SNPs.

The SNPs were detected between each IMP-4-producing strain and the reference K1 strain (Figure 3). The results showed that the sputum isolate K3 from patient 3 was the most distinct one differing from the other eight isolates by 239–275 SNPs. The isolate K9 from the duster cloth was the second distinct one differing from the other seven isolates (except for K3) by 10–36 SNPs. The first isolate K1 from patient 1 and the sputum and blood isolates K2 and K4 from patient 2, respectively, showed the closest relationship (0–2 SNPs). The synonymous and non-synonymous SNPs between each pair of strains are shown in detail in Table 2. Since they were distinctly different from the other strains, the SNPs of the K3 and K9 strains are not shown.

**Table 2:** SNPs detected among the isolates in this study.

**SNPs**	**K1**	**K3**	**K4**	**K5**	**K6**	**K7**	**K9**	**SNP change**	**Codon change**	**Amino acid change**	**Mutation type**
1	T	T	T	T	T	A	T	T<->A	AAT<->ATT	N<->I	Non-synonymous
2	T	T	T	T	T	G	T	T<->G	AAT<->AAG	N<->K	Non-synonymous
3	A	A	A	A	A	C	A	A<->C	ATA<->CTA	I<->L	Non-synonymous
4	C	G	G	G	G	G	G	C<->G	CAG<->GAG	Q<->E	Non-synonymous
5	T	C	C	C	C	C	C	T<->C	TTG<->TCG	L<->S	Non-synonymous
6	T	T	T	T	A	T	T	T<->A	GCT<->GCA	A<->A	Synonymous
7	A	A	A	G	A	A	A	A<->G	AGC<->GGC	S<->G	Non-synonymous
8	G	G	G	A	G	G	G	G<->A	AGC<->AAC	S<->N	Non-synonymous
9	C	C	C	A	C	C	C	C<->A	AGC<->AGA	S<->R	Non-synonymous
10	A	T	A	A	A	A	A	A<->T	ACA<->TCA	T<->S	Non-synonymous
11	C	C	C	A	C	C	C	C<->A	ACA<->AAA	T<->K	Non-synonymous
12	A	T	A	A	A	A	A	A<->T	ACA<->ACT	T<->T	Synonymous
13	C	A	C	C	C	C	C	C<->A	GCG<->GAG	A<->E	Non-synonymous
14	G	G	G	T	G	G	G	G<->T	GCG<->TCG	A<->S	Non-synonymous
15	G	G	G	A	G	G	G	G<->A	GGA<->GAA	G<->E	Non-synonymous
16	T	G	T	G	T	T	T	T<->G	TTA<->GTA	L<->V	Non-synonymous
17	A	G	A	G	A	A	A	A<->G	AAA<->AAG	K<->K	Synonymous
18	A	G	A	G	A	A	A	A<->G	ATG<->GTG	M<->V	Non-synonymous
19	A	G	A	G	A	A	A	A<->G	ATC<->GTC	I<->V	Non-synonymous
20	T	T	T	T	T	G	T	T<->G	GTG<->GGG	V<->G	Non-synonymous
21	A	A	A	A	A	C	A	A<->C	GAG<->GCG	E<->A	Non-synonymous
22	A	A	A	A	A	C	A	A<->C	GAA<->GCA	E<->A	Non-synonymous
23	T	T	T	T	T	G	T	T<->G	TTT<->TTG	F<->L	Non-synonymous
24	A	A	A	A	A	C	A	A<->C	AAG<->CAG	K<->Q	Non-synonymous
25	C	T	T	T	T	T	T	C<->T	ACC<->ACT	T<->T	Synonymous
26	C	A	A	A	A	A	A	C<->A	GAC<->GAA	D<->E	Non-synonymous
27	C	A	A	A	A	A	A	C<->A	ACG<->AAG	T<->K	Non-synonymous
28	C	C	C	A	C	C	C	C<->A	CTG<->CTT	L<->L	Synonymous
29	T	G	T	T	T	T	T	T<->G	GAA<->GCA	E<->A	Non-synonymous

*SNPs are highlighted in red*.

*Only synonymous and non-synonymous mutation were shown*.

Based on the SNPs, a maximum-likelihood phylogenetic tree was reconstructed, and the 9 strains were partitioned into three clades. Clade 1 comprised seven strains (K1–K2 and K4–K8), whereas clade 2 and 3 consisted of only one strain each: K3 and K9, respectively (Figure 3). The SNPs tree showed that the hand of the nurse, but not the duster cloth, might have participated in this nosocomial infection.

## Discussion

In this report, we describe a hospital infection of the recently emerged IMP-4 positive CRKP in a tertiary care hospital in China. The IMP-type metalloenzymes have been reported worldwide, with a higher prevalence in southern Europe and Asia, resulting in problems regarding therapy and control.

The dissemination of *bla*IMP-4 is associated with the MLST type. IMP-4-producing *K. pneumoniae* have been reported in different countries, and belong to various kinds of MLST types, including ST105, ST263, and ST265 (27, 28). Our data indicated that all the nine IMP-4-producing *K. pneumoniae* strains belong to the same type, ST2253, which was different from the types previously reported. Carbapenemase IMP is often distributed among multiple STs in *K. pneumoniae*, especially some rare STs (13). The goeBURST algorithm (http://www.phyloviz.net/) was used to infer the evolutionary relatedness of the STs. The novel ST2253 was not related to any known endemic/epidemic IMP-4 producing CR-KP clones that had been reported in China. ST2253 has no single-locus variant and double-locus variants. We reported the first clonal dissemination of IMP-4-producing CR-KP ST2253 clone and demonstrated that this new ST is a potential high-risk clone that needs close attention.

The conventional molecular typing approaches, such as PFGE and MLST, have been used to enhance the prevention and control of nosocomial pathogen outbreaks or dissemination (29). However, with the universal application of the next generation sequencing, WGS diversity has become a popular and important molecular typing method with a higher typing resolution compared with the methods of PFGE and MLST (30) and enabled a detailed understanding of how the transmission occurs and the pathogen spreads. Understanding the epidemiological and molecular features of the CRKP population can be helpful to control their dissemination. In this study, we used WGS to study the IMP-4 producing *K. pneumoniae* infection clone that was assigned to a new sequence type (ST2253). Although the 9 IMP-4-producing *K. pneumoniae* strains possessed similar PFGE patterns and had the same sequence type, the SNP variations in the whole genome allowed us to divide the strains into three clades (Figure 3). Additionally, the SNPs tree suggested that the hand of the nurse may have contributed to the spread of these organisms within the hospital. The sampling times of these patients and the environment were close, suggesting that a dissemination event occurred within a short period of time. Although the sampling time of the strain K3 was very proximate to the strains in clade 1, this strain (K3) was still not considered to participate in this transmission. The isolate K3 was very distinct from the other isolates in the analysis. It is particularly interesting that it was so distant from the isolate K7 obtained from patient 3. The reasons behind this intra-host diversity need further exploration, especially in such a young patient where a long term colonization is not feasible.

Controlling the dissemination of multidrug-resistant pathogens remains one of the major public health challenges. In order to prevent further spread, we conducted strict infection control measures covering the patients and medical staff in December 2018, including stopping the admission to the NICU, terminal disinfection of the environment and effective training of all the NICU staff. The patients on “high risk” wards, defined as those wards having the same medical staff and extensive patient exchange with the ones where positive patients stayed, were screened for CRKP once a week, while the patients overlapping with positive patients on the ward were screened twice a week. Our research showed that the strain was detected on the personnel hands that might have contributed to the transmission by sequence data, and we trained all the medical staff to strictly enforce hand hygiene. We also increased the number of staff and controlled the admissions to decrease the patient/staff ratio.

Since we implemented these procedures, the repeated screenings of the medical staff did not detect any CRKP. Likewise, the strain was neither detected among patients from other wards, nor from environmental sources in this NICU. After active and effective treatment, the three infants all have a good prognosis. This nosocomial infection in the NICU may have been controlled.

However, there are still some limitations in our study. First, we still do not know how the IMP-4 was introduced into the unit. Since we do not undertake routine screening for multi-resistant gram-negative bacteria, it is possible that the CRKP strain was previously present but undetected. A more complex route might result in the transmission, an intermediate patient or an environmental source. We screened the samples taken from the rectal swabs and breast milk of the three neonates' mothers, and no CRKP were isolated, which suggested that the CRKP strains were not initially from their mothers. Secondly, we suspect that the CRKP strains in this study represent a novel ST, and that autochthonous clones are locally acquiring plasmids carrying the *bla*IMP-4. Due to our study mainly focused on the hospital infection tracking and control of CR-KP clinical clones, we only found the nine isolates carried IncN plasmid but did not carry out detailed analysis of plasmid. The *bla*IMP-4 gene is often integrated in broad-host-range conjugative plasmids and is transferred between different Gram-negative bacilli (31). Plasmid-borne *bla*IMP-4 has been sporadically reported in different Gram-negative bacilli in China but only a few studies have reported the complete sequence of *bla*IMP-4-harboring plasmids (32, 33). Further plasmid investigation may help to understand the genomic features of this bacterial pathogen in the future research. Another limitation of this study is represented in the small number of the investigated isolates and the local nature of the infection incident; thus, a considerably larger series of isolates that are recovered over the course of different CRKP outbreaks, preferably in more than one hospital, would be required to increase our understanding of the epidemiology of CRKP and inform the discriminatory value of the SNP variations.

## Conclusion

We reported the first clonal dissemination of IMP-4-producing CR-KP ST2253 clone and demonstrated that this new ST was not related to any known endemic/epidemic IMP-4 producing CR-KP clones that had been reported in China, which is a potential high-risk clone that needs close attention. WGS successfully tracked and controlled the local infection of the novel ST2253 that prevented a more serious nosocomial infection. IMP might spread without attracting attention, especially in the NICU environment in which the hands of nurses and doctors may be the diffusion reservoirs of IMP-4-producing strains. Using surveillance cultures and initiating strict hygiene procedures are mandatory for the prevention and early detection of CRKP or other *Enterobacteriaceae* in the units where high-risk patients receive care.

## Data Availability Statement

The data analyzed in this study is subject to the following licenses/restrictions: Permission to use the data sets was obtained under strict conditions that it should be used for academic purposes only, and not be made available to the public or any other organizations. Requests to access these datasets should be directed to slyywsw@163.com.

## Ethics Statement

The studies involving human participants were reviewed and approved by Shandong Provincial Hospital affiliated to Shandong First Medical University, Biomedical Ethics Committee. Written informed consent to participate in this study was provided by the participants' legal guardian/next of kin.

## Author Contributions

YB and YJ conceived the study and wrote the manuscript. YW designed the experiments. YB and CS performed the laboratory experiments. YB, YJ, and YH analyzed the data, contributed to preparing the final version of the manuscript, and approved the final manuscript. All authors contributed to the article and approved the submitted version.

## Funding

This work was supported by the Natural Science Foundation of Shandong Province (grant number ZR2016HB44) and Focus on Research and Development Plan in Shandong Province (grant number 2016GSF201078).

## Conflict of Interest

The authors declare that the research was conducted in the absence of any commercial or financial relationships that could be construed as a potential conflict of interest.

## Publisher's Note

All claims expressed in this article are solely those of the authors and do not necessarily represent those of their affiliated organizations, or those of the publisher, the editors and the reviewers. Any product that may be evaluated in this article, or claim that may be made by its manufacturer, is not guaranteed or endorsed by the publisher.
