# Supplementary material for: Using Whole Genome Sequencing to Trace, Control and Characterize a Hospital Infection of IMP-4-Producing Klebsiella pneumoniae ST2253 in a Neonatal Unit in a Tertiary Hospital, China
Source: Front Public Health. 2021 Dec 15;9:755252. doi: 10.3389/fpubh.2021.755252 (PMC8715938; doi:10.3389/fpubh.2021.755252)
Supplement: Supplementary file 1 [file Data_Sheet_1.PDF]

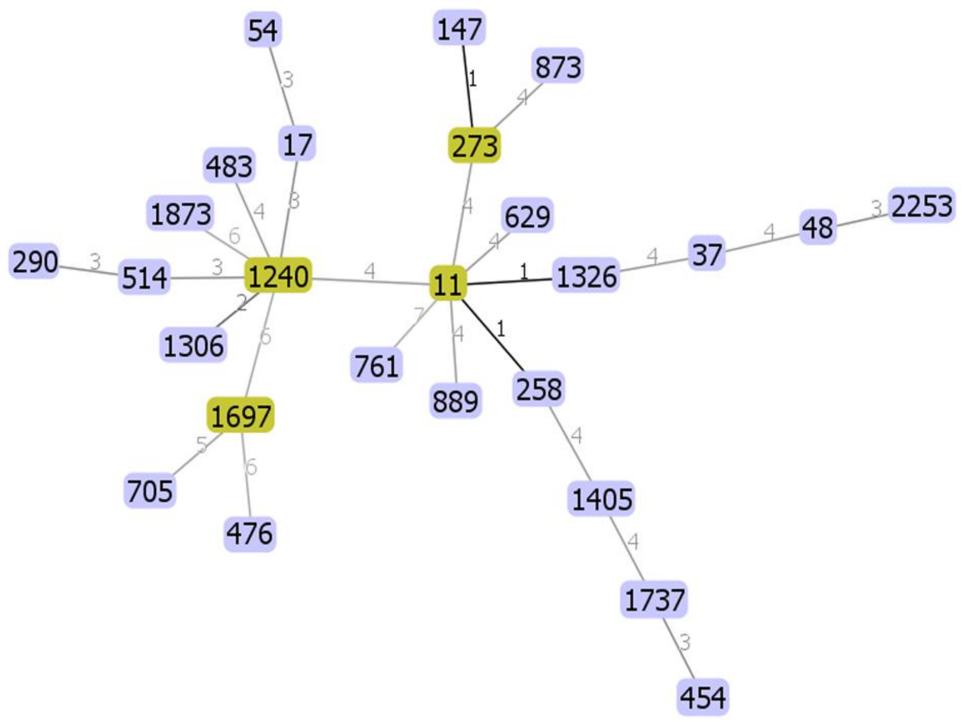

Supplementary Figure 1. MLST based minimum spanning tree.
PHYLOViZ-generated minimum-spanning tree is based on the allele number matrix of the gene loci included in the *K. pneumoniae* MLST scheme. Numbers in the circles indicate the MLST sequence type. Numbers along the linking lines indicate the absolute distance.
